# Editorial: Human Perception of Environmental Sounds

**DOI:** 10.3389/fpsyg.2021.714591

**Published:** 2021-06-28

**Authors:** Francesco Aletta, Bert De Coensel, PerMagnus Lindborg

**Affiliations:** ^1^Institute for Environmental Design and Engineering, The Bartlett, University College London, London, United Kingdom; ^2^WAVES Research Group, Department of Information Technology, Ghent University, Ghent, Belgium; ^3^School of Creative Media, City University of Hong Kong, Hong Kong SAR, China

**Keywords:** soundscape, perception, environment, acoustics, urban planning, health, design, noise reduction

## 1. Introduction

Environmental sounds are a key component of the human experience of a place as they carry meanings and contextual information, together with providing situational awareness. They have the potential to either support or disrupt specific activities as well as to trigger, to inhibit, or simply to change human behaviors in context. The experience of acoustic environments can result in either positive or negative perceptual outcomes, which are in turn related to well-being and Quality of Life. In spite of its relevance to the holistic experience of a place, the auditory domain is often not given enough prominence in environmental psychology studies. Environmental sounds are typically considered in their negative perspective of “noise” and treated as a by-product of society. However, the research (and practice) focus is gradually shifting toward using environmental sounds as mediators to promote and enrich communities' everyday life. Designers explore how natural sounds can be mixed into urban life.

While there is a lot of research happening in this area, the underlying mechanisms connecting environmental sounds, the physical and social context where they occur, and their perceptual effects on users, are still not fully understood (Axelsson et al., 2019). Furthermore, when exploring the aforementioned relationships, more challenges arise in terms of psychometrics and ecological validity of the methodologies involved. All such issues need to be addressed by researchers and practitioners of the built environment. For this reason, a broad spectrum of submissions was invited for this Research Topic. Article types ranged from conceptual analyses, to reviews and research papers. The studies presented here dealt with the characterization and perception of single environmental sounds or complex acoustic environments, as well as their management and design implications for the urban realm. The focus could either be on theoretical aspects (e.g., relationships between sounds and psychological and physiological aspects) or methodological aspects (e.g., protocols and procedures to gather objective and subjective data).

## 2. Research Themes

Considering the broad scope of the call for papers, the topics and research questions addressed by the submissions we received were very diverse. Looking retrospectively at them, we tried to identify common themes and eventually clustered them under four main categories. These were: Soundscape theory; Soundscape for health and well-being; Sound perception in urban environments; and Soundscape design. As we see, soundscape is a recurring concept; this is unsurprising considering how “perception”—which was the core aspect of this Research Topic— is an intrinsic aspect in soundscape studies (Kang et al., 2016). The term soundscape itself was standardized and defined as an “acoustic environment as *perceived* and/or understood by a person, or people, in context” (ISO, 2014). The standardization process for other methodological aspects is still in progress and is expected to be informed by the intense activity currently taking place both in soundscape research and practice.

### 2.1. Soundscape Theory

While the first harmonization efforts in soundscape studies started more than a decade ago, soundscape theory *per se* could still be considered at an early stage of development for many aspects. If consensus has been found on some basic definitions and frameworks, there is still a lot of debate around methodological approaches, as well as theoretical models underpinning the soundscape concept itself, and how it relates to human psychology and physiology. Thus, contributions to this particular research strand were particularly welcome to advance the scientific conversation on these issues. Chen and Ma synthesized data from semi-structured interviews with 75 participants using a Grounded Theory approach and proposed a conceptual model to define and characterize healthy acoustic environments. Fiebig et al. proposed a conceptual paper about the application of emotion theory to soundscape studies. Their analysis revolves around three main themes, namely: the effect that the acting of collecting soundscape itself can have on people's emotional response to an acoustic environment; whether the affective qualities of a soundscape are actually consciously accessible to people in the first place; and whether it is possible for people to separate the emotion related to a sound environment from affective predisposition. Lionello et al. worked on a large-scale soundscape survey dataset collected in accordance with the ISO/TS 12913-2:2018 and explored how people interpret the Likert scale metrics associated with that soundscape data collection instrument in psychometric terms.

### 2.2. Soundscape for Health and Well-Being

On the one hand, many aspects of the negative influence of noise on people's health and well-being are well-established through research over the past several decades. For example, sustained exposure to noise near airports or high-density road traffic, even at levels well within legal regulations, has been linked to higher incidence of a range of cardiac illnesses, and overly reverberant classrooms blur phoneme perception and slow down children's learning of language. This knowledge and concerted efforts to enforce regulations have gradually come to influence urban planning (European Parliament and Council, 2002) and construction (Department for Education, 2003). On the other hand, positive effects of sound and soundscape have only more recently moved into focus (Aletta et al., 2018). This is investigated in four articles of the Research Topic. Zhou et al. conducted an experiment with 70 hospital inpatients. Participants listened to soundscape and music recordings through a virtual reality headset, and the researchers measured physiological markers on psychological stress recovery. Using a similar experimental setup, Benfield et al. presented visual images of natural parks while manipulating the soundscape by adding different types of extraneous noise. Responses from 229 volunteers were analyzed with a time series approach. Eqlimi et al. measured the effect of different kinds of ecologically valid noise, such as highway traffic or multi-talker babble, on a learning task. They linked noise type to specific neural correlates that have a negative impact on overall attentive state and capacity to decode spoken language. Gasco et al. adopted a big-data approach: they used geo-referenced social media images from Flickr to characterize the city soundscape of London and built a model with socioeconomic variables, official noise exposure levels, and the soundscape estimated from social media as indicators to predict health outcomes for the population. Ratcliffe offered a comprehensive narrative literature review on the growing research area investigating the relationships between soundscapes, the experience of nature, and restorative environments, which is of theoretical interest for health-related studies.

### 2.3. Sound Perception in Urban Environments

While the previous experimental studies employed relatively controlled audio stimuli, the next three articles chart complex acoustic environments in built-up, urban spaces, with a bearing on architecture and urban planning. Taghipour et al. set up a laboratory listening experiment and explored how conventional room acoustics parameters would perform in predicting the perceived acoustic comfort in outdoor proximity spaces (e.g., courtyards, balconies) of residential buildings. In Versümer et al. authors surveyed a large number of participants who were asked to recall and describe low-level sounds in everyday situations. The researchers identified a range of sound source types, and explored their impact of on annoyance, valence, arousal, and mental fade-out ability, along with individual and demographic predictors. Lenzi et al., report an observational study during several months of the initial pandemic lockdown in the Basque Country. Pleasantness, eventfulness, and sound source type were analyzed to yield a picture of how people and animals reacted to the extraordinary circumstances in terms of their acoustic communication, as well as people's use of different modes of transport and outdoor-indoor behavior.

### 2.4. Soundscape Design

The fourth group is exemplary of applied sound perception research. The articles described how the sonic material was deliberately varied and perceptions measured while at the same time the authors had an aesthetic design goal with their work. Rajguru et al. prepared a mini-review about challenges and opportunities in spatial sound perception and soundscape studies, with a focus on augmented and virtual reality methodologies. Cuadrado and collaborators conducted an experimental study on 253 primary school children where some of the sonic elements of an audio story were marked through spatial design. In a mixed-methodology approach, emotional responses were measured with electrodermal resistance, and the children self-reported immersion and mental imagery. Finally, Trudeau et al. reported a case study at a public plaza in Montreal. Visitors evaluated the perceived quality of the sonic environment, where three different designs of a water feature alternated.

## 3. Concluding Remarks

While the four themes discussed above certainly do not cover the full range of questions being debated in the context of soundscape studies, they do detect a few "hot topics" and areas of interest for researchers and practitioners in the field. The psychological theory underpinning the environmental sounds perception processes could still be considered (at least) as evolving. There is a clear interest in making a connection with health and well-being frameworks and also an outlook toward design and co-creation of open public spaces. Virtual reality techniques are now commonly used in perceptual experiments, together with onsite surveys and the analysis of “big data” from public sources. Going forward, it will be essential to include all possible stakeholders in the debate: the public, researchers, practitioners, artists, and professionals with different skills and expertise. This will help testing new hypothesis and triangulate methodologies and results.

## Author Contributions

All authors listed have made a substantial, direct and intellectual contribution to the work, and approved it for publication.

## Conflict of Interest

The authors declare that the research was conducted in the absence of any commercial or financial relationships that could be construed as a potential conflict of interest.
